# Isolated sublingual hematoma post internal carotid artery stenting for internal carotid artery stenosis in high-risk patients as uncommon and rare misadventure: A case report and review of literature

**DOI:** 10.1016/j.ijscr.2022.107070

**Published:** 2022-04-11

**Authors:** Ahmed Mousa, Bassam A. Khalil

**Affiliations:** a Department of Vascular Surgery & Endovascular Therapy, Al-Hussain University Hospital, Faculty of Medicine for Males, Al-Azhar University, Darrasa, Cairo, Egypt; b Unit of Vascular Surgery, Department of General Surgery, King Faisal Specialist Hospital and Research Centre (KFSH&RC), Riyadh, Saudi Arabia

**Keywords:** High-risk patient, Symptomatic internal carotid artery stenosis, Internal carotid artery stenting, Sublingual hematoma, Case report

## Abstract

**Introduction and importance:**

Isolated sublingual hematoma is a rare complication seen in trauma, severe uncontrolled hypertension, dental operations, bleeding diathesis, and the use of dual antiplatelet and anticoagulant agents. In advanced and neglected cases, a sublingual hematoma may interfere with the patient's airway, causing suffocation and fatal airway obstruction. Our objective was to present a case of iatrogenic isolated sublingual hematoma in a 70-year-old business man, heavy smoker with a history of hypertension. Furthermore, to report the literature review, and to organize treatment strategies to reduce the rate of progression of the hematoma. In addition to recommend or advice a strategic plan to prevent this complication during carotid stenting.

**Case presentation:**

This case report has been reported in line with the SCARE Criteria. We represented a case report of an iatrogenic isolated mild/moderate sublingual hematoma in a 70-year-old business man. This hematoma developed as one of the rare complications of endovascular internal carotid artery revascularization because of injury to the sublingual branch of the lingual artery during wire manipulations or its advancement. This hematoma was treated by conservative treatment without any intervention.

**Clinical discussion:**

Immediately after internal carotid artery stenting procedure, the patient developed a sudden onset of painful swelling in the floor of the mouth. The hematoma showing ecchymotic submucosal swelling underneath the tongue in the floor of the mouth. If it became enlarged, it may push the tongue against the palate and blocking the airway, causing serious airway obstruction. Fortunately, the swelling is isolated (has no extension to any side) and limited to the root and middle third of the tongue but, not extending to its tip. Its development most probably due to injured atherosclerotic sublingual branch of the lingual artery during the procedure.

**Conclusions:**

The first step in management should be prompt airway management. Conservative treatment took place without any further intervention. To date, there is no consensus about the management regarding the hematoma itself. Mostly, clinicians start with observation for spontaneous resolution. When conservative treatment is not appropriate, surgical intervention must be performed. However, electively secure the airway is the main objective for treatment.

## Highlights

• We presented a case report of iatrogenic sublingual hematoma as a complication of internal carotid artery stenting.
• The first step in management should be prompt airway management.
• As the patient was stable without any respiratory and airway compromise, conservative treatment took place without any further intervention, by closed observation and watchful waiting.
• When conservative treatment is not appropriate or failed, surgical intervention must be performed.
• To date, there is no consensus about the management of the hematoma itself.
• Finally, and according to our best of knowledge, this is the first and a unique case that reported an isolated sublingual hematoma following internal carotid artery stenting.

## 1 Introduction

We presented a case report of iatrogenic sublingual hematoma as misadventure and a rare complication of endovascular internal carotid artery stenting for treating patient with symptomatic internal carotid artery stenosis. Sublingual hematoma is considered as a rare and potentially fatal complication that may develop after carotid artery stenting (CAS). The tongue is a highly vascular structure with extensive anastomotic network. Therefore, there is a potential risk of hemorrhage and consequently hematoma formation following local trauma or iatrogenic injury during any related procedures [1]. The rate of complications after CAS varies from 3.0% to 4.4%, and mostly include ischemic stroke, intracranial hemorrhage, or groin complications [2]. Rapid tongue enlargement secondary to hematoma formation may lead to a life-threatening respiratory and airway obstruction. However, prompt recognition and urgent management is mandatory. There were a variety of the etiological factors for development of sublingual hematoma. Its causes range from traumatic to spontaneous variety with or without the use of anticoagulants. Traumatic sublingual hematoma most commonly occurs as a result of motor vehicle accidents, child abuse, assault, and seizures. Inherited coagulopathy or anticoagulant therapy is usually the causes of spontaneous sublingual hematoma [3], [4], [5], [6]. Furthermore, patients with vascular diseases and atherosclerosis of the lingual arterial system have increased risk for sublingual hematoma. Mainly due to vessel tortuosity, fragility, and possible shearing [7]. The management of sublingual hematoma is controversial regardless of etiology. Starting from conservative management up to tracheotomy. However, early recognition of the initial presentation, its possible cause and the relevant anatomy is critical for the proper management of such condition [8]. In the current study we reported a case of iatrogenic uncommon and rare complication of internal carotid artery stenting. We highlighted the importance of endovascular skills when performing carotid intervention to avoid or at least minimize interventional complications as well as the significance of closed postoperative monitoring of patients undergoing carotid artery stenting. However, conservative treatment took place without any further intervention, by closed observation and watchful waiting for increasing the size of the sublingual hematoma, and subsequently and airway obstruction, which may develop a fatal suffocation. The first procedure in the management should be prompt airway patency. To date, there is no consensus agreement about the proper management of sublingual hematoma. Moreover, and usually most clinicians start with observation and follow up, in the hope of spontaneous resolution of such hematoma. When conservative treatment is not appropriate or failed, surgical intervention must be performed, in addition the treatment of the precipitating or causative factor. However, the main goal is to electively secure the airway. Furthermore, and in emergency airway obstruction, surgical tracheotomy must be performed to electively secure the airway. However, and according to our best of knowledge, this is the first and a unique case that reported an isolated sublingual hematoma following internal carotid artery stenting. This case report has been reported in line with the SCARE Criteria [9].

## 2 Presentation of case

**Fig. 1:**
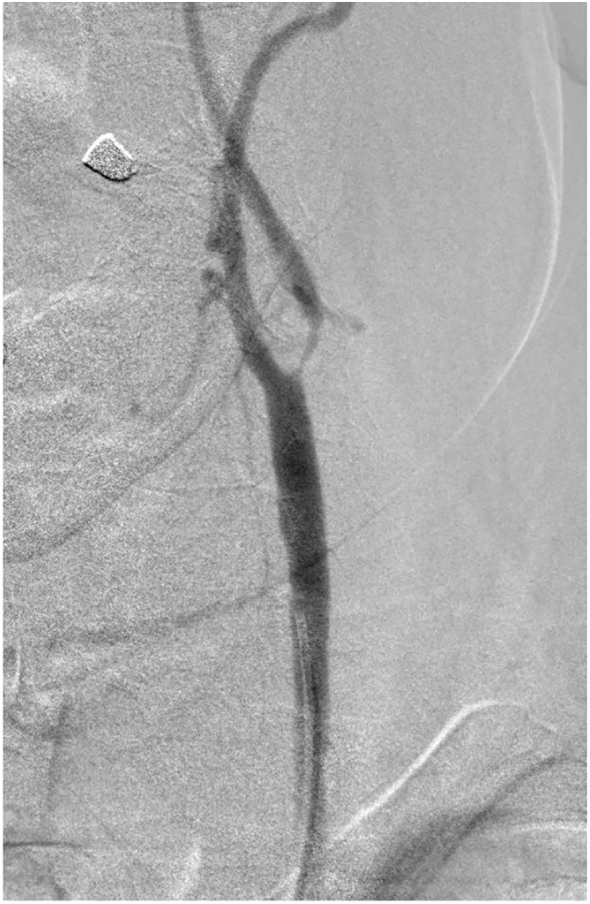
DSA showing tight left internal carotid artery stenosis. Foot note: DSA, Digital Subtraction Angiography.

**Fig. 2:**
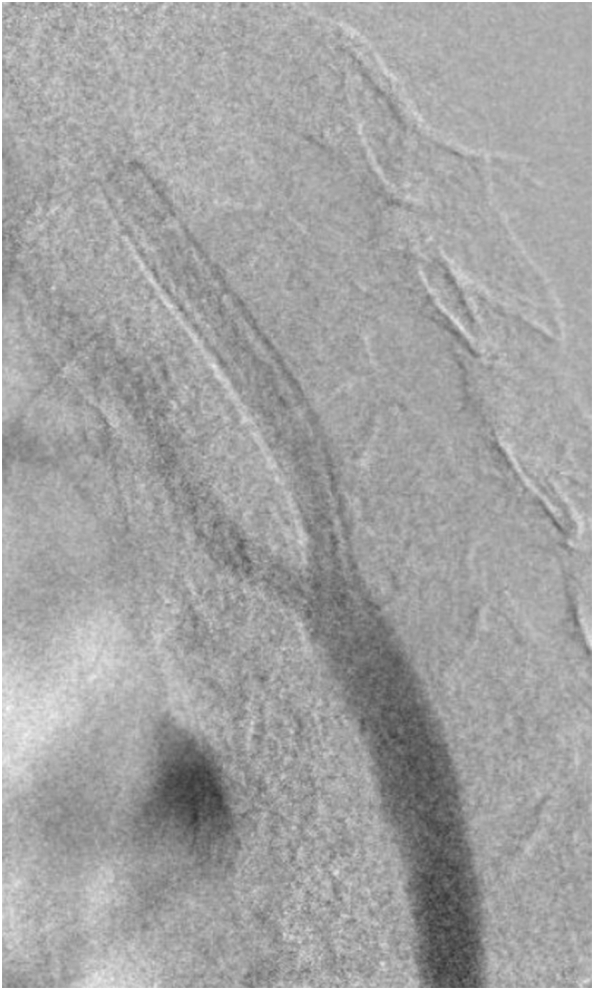
DSA showing self-expandable stent of the left ICA. Foot note: DSA, Digital Subtraction Angiography; ICA, Internal Carotid Artery.

**Fig. 3:**
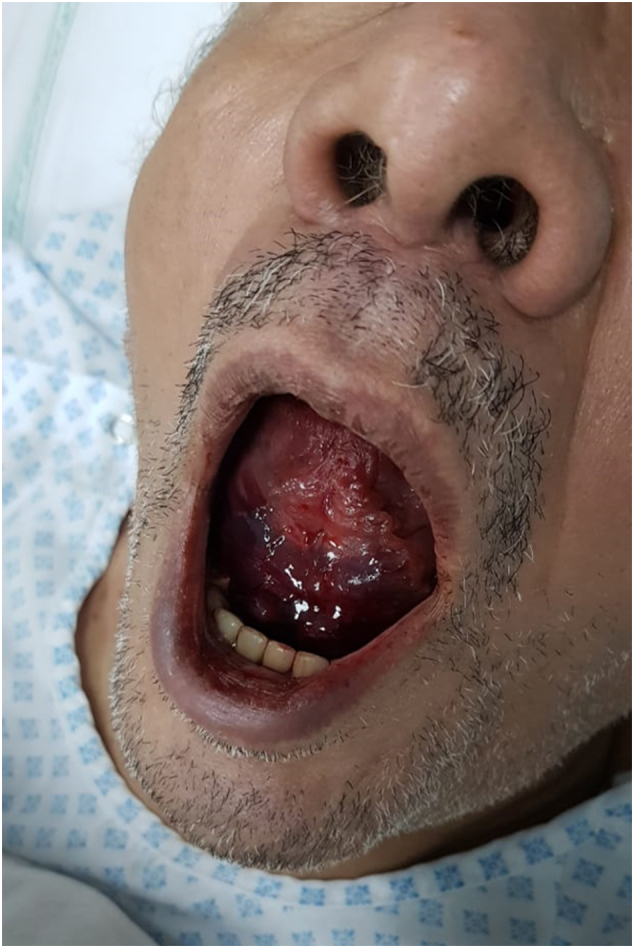
Sublingual hematoma measuring about 4 × 5 cm.

**Fig. 4:**
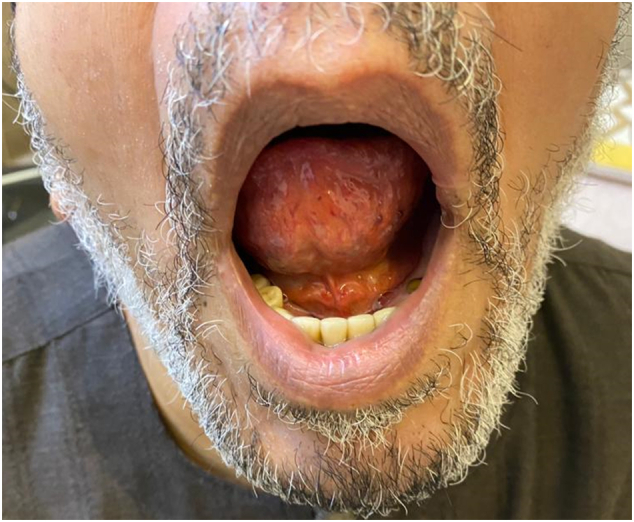
Completely resolved sublingual hematoma within one week of conservative treatment.

This work has been reported in line with the SCARE Criteria [9]. A 70-year-old male, business man, heavy smoker, and a known case of hypertension, was referred to our hospital. The patient was heavy smoker with a history of hypertension. He referred to us from other hospital clinician with a history of repeated transient ischemic attacks (TIA) throughout a year back. Some of the TIA was associated with some sort of loss of consciousness for a short period of time. However, the patient got two cerebrovascular stroke (CVS), three months and one month respectively, before referral. The first one resulted in left-sided hemiparesis that recovered completely with conservative medical treatment, in addition to a condensed course of physiotherapy. The second CVS affecting the right side, and resulted in slurred speech. Bilateral carotid computed tomography angiography (CTA) scanning was performed. It revealed right and left internal carotid artery (ICA) stenoses, 86% and 75% respectively. We decided to stent the right sided first. The stenting procedure was performed successfully without any complications. The patient was appointed for a second visit after a six month period to treat the left-sided obstruction. Then he admitted after control of hypertension on an outpatient basis. If the patient was on antiplatelet therapy or on oral anticoagulants, the former stopped 24 h, while the later stopped at least 48 h prior to the procedure. General anesthesia was taking place. However, the procedure was performed at King Faisal Specialist Hospital and Research Center (KFSH&RC), Riyadh, Saudi Arabia, by an experienced and board certified vascular and endovascular surgeon. Diagnostic digital subtraction angiography (DSA) was performed. It revealed about 90% tight left ICA stenosis, starting from its origin (carotid bifurcation) upwards (Fig. 1). An 80 × 40 mm self-expanding carotid stent (Abbott XACT) was deployed (Fig. 2). The first and the second procedures were adopted using an embolic cerebral protection device (Emboshield NAV6; Abbott Laboratories, North Chicago, IL). However, after left ICA stenting, in the immediate postoperative period, the patient complained of sudden onset of painful tongue swelling, associated with sublingual swelling, drooling, sore throat, dysphagia, and difficulty in speech, which is the patient's main concern. Oral examination revealed mild to moderate tongue swelling associated with an isolated sublingual hematoma measured about 4 × 5 cm (Fig. 3). It is entirely situated in the floor of the mouth, and firm to palpation. Associated with ventral tongue ecchymosis extending from the base of the tongue up to its middle third, and not extended to its tip. Neck examination wasn't considered significant for swelling in the submental region. Complete blood count and coagulation profile were all performed and including activated partial thromboplastin time (APTT), bleeding and clotting times, were also performed to exclude bleeding complications from the given anticoagulants. Moreover, the patients' airway was patent without any respiratory compromise with normal oxygen saturation. We consult our Ear Nose Throat (ENT) surgical team that performed oral examination and direct laryngoscopy to visualize the focal folds and ensure patency of the laryngeal air way. They advised to treat the patient conservatively, as the tongue swelling and its sublingual hematoma is not large enough to cause either respiratory compromise or airway obstruction. The patient underwent closed observation with respiratory monitoring, airway patency, as well as the oxygen saturation level. In addition to broad spectrum antibiotic and antiedema therapy. On the 2nd postoperative day, the hematoma was stable, not increased in size. The airway was also patent without any respirator compromise. Postoperatively, and on the 3rd day the sublingual hematoma was observed to decrease in size with improvement of the patient's speech, no dysphagia, or sore throat. The patient was discharged and instructed to follow up within the next week, where the sublingual hematoma has been resolved and completely disappeared (Fig. 4). Perioperatively, the patient received dual antiplatelet agents, in the form of clopidogrel 75 mg/day, in addition to aspirin 81 mg/day, 7–10 days before carotid stenting and one month thereafter. During the endovascular procedure, the patient received unfractionated heparin (5000–10,000 IU) intravenous bolus to maintain an activated partial thromboplastin time (APTT) > 250 s.

## 3 Discussion

Bleeding into the sublingual space may elevate the tongue and increase the dead space in the floor of the mouth resulting in a non-infectious life-threatening upper airway obstruction. This picture was first reported in 1976, and known as pseudo-Ludwig's phenomenon [10], [11]. Sublingual hematoma may be seen in trauma, tongue bite, biopsies at the level of the mouth floor, dental/osseodental procedures, intraoral suturing, or in patients with bleeding disorders. However, it may have described as a complication of dual antiplatelet and anticoagulant therapy, where there is a tendency of spontaneous bleeding [12], [13], [14], [15]. However, it may develop as a result of severe uncontrolled hypertension, as well as atherosclerotic disease affecting the lingual artery. All of these factors may interfere with the patency of the upper airway and may cause respiratory compromise. Airway obstruction leads to secondary pulmonary edema as well as anoxic brain damage [16], [17], [18], [19]. However, early complaint of sore throat, may be the first presentation, particularly in patients receiving dual antiplatelet and anticoagulant therapy [14], [15].

Although, there are no literature reports regarding the application of either ice or a cold packs or adrenalin packs. They may be considered accepted methods of causing local vasoconstriction of the bleeding vessel/s but, not reducing the size of the hematoma. They may be used under the chin and applied for 10 to 20 min at a time. The periprocedural risk of stroke or death following carotid artery stenting (CAS) was estimated as 4.7% (range 2–9%) from a Cochrane review of more than 5000 CAS procedures [20], [21]. The most common complications associated with CAS are thromboembolic events, as it accounts for 4.1% to 6.5% [22]. In this case study, we reported a case of an isolated iatrogenic sublingual hematoma following endovascular ICA stenting. We suggested that this sublingual hematoma may developed because of injury to the sublingual branch of the lingual artery that anastomosing with the submental and the incisive arteries, both are branches of the facial and the inferior alveolar arteries respectively [23]. Our patient was discovered to have sublingual hematoma in the immediate postoperative period. His airway was patent with normal oxygen saturation. However, post-stenting, the patient was given routinely a combination of antiplatelet therapy in the form of acetylsalicylic acid (aspirin) 81 mg/day, and clopidogrel 75 mg/day. We were afraid of that hematoma increased in size. Successful conservative management took place as recommended without any surgical intervention. This is done by the use of a custom soft tissue guard to protect the patient's airway and to assure its patency. Closed observation was carried out to follow up the patient and to maintain the patency of the airway. Iatrogenic injury of the external carotid artery (ECA) branches is a potentially fatal complication of endovascular carotid procedures secondary to wire manipulation in the small vessels. In the published literature form the University of Buffalo, they reported that 1000 patients underwent carotid endovascular procedures. Only four of them underwent vessel perforation, including the fascial, lingual and occipital arteries. However, the cause in the fourth case was developed as a result of predilatation balloon, inflated in the ascending pharyngeal artery [24]. Other reported an overwhelming sublingual hematoma following osseointegrated transplant situated in the mandible anteriorly [25].

## 4 Conclusions

We reported this case to endorse the importance of postoperative care of carotid artery stenting procedures and to introduce such rare yet a potentially fatal complication. The first line management of isolated sublingual hematoma, should be prompt airway patency, and it must be considered as the main goal for treatment. To date, there are no reported guidelines regarding the management of such complication. However, most clinicians start their management conservatively, in the hope of spontaneous resolution. When conservative treatment is not appropriate, surgical intervention must be performed. Finally, this is the first reported isolated sublingual hematoma after carotid artery stenting according to the best of our knowledge. On failure of conservative treatment and watchful waiting, surgical intervention in the form of tracheotomy must be adopted to electively secure the airway. In the future, and to avoid this complication, we recommend gentle manipulations and exchange of the guide wires during carotid artery stenting. However, empirical administration of unfractionated heparin during the procedure must be avoided, and provided according to the body weight or it may be given at a suboptimal dose.

## Informed consent

Written informed consent was obtained from the patient for publication of this case report and accompanying images. A copy of the written consent is available for review by the Editor-in-Chief of this journal on request.

## Provenance and peer review

Not commissioned, externally peer-reviewed.

## Ethical approval

The authors declare that, they obtained permission from the ethics committee in our institutions.

## Funding

None declared.

## Guarantor

Ahmed Mousa.

## Research registration number

None.

## CRediT authorship contribution statement

**Ahmed Mousa:** Data curation, Formal analysis, Investigation, Methodology, Validation, Writing, original draft, Writing, review and editing.

**Bassam A. Khalil:** Data curation, Formal analysis, Investigation, Methodology, Validation, Writing, original draft, Writing, review and editing.

## Declaration of competing interest

None declared.
